# Muographic monitoring of the volcano-tectonic evolution of Mount Etna

**DOI:** 10.1038/s41598-020-68435-y

**Published:** 2020-07-09

**Authors:** D. Lo Presti, F. Riggi, C. Ferlito, D. L. Bonanno, G. Bonanno, G. Gallo, P. La Rocca, S. Reito, G. Romeo

**Affiliations:** 1 Department of Physics and Astronomy “E. Majorana”, University of Catania, Catania, Italy; 2 INFN, Sezione di Catania, Via S. Sofia 64, 95123 Catania, Italy; 3 Department of Biological, Geological and Environmental Sciences, University of Catania, Catania, Italy; 4 INAF, Osservatorio Astrofisico di Catania, Via S. Sofia 78, 95123 Catania, Italy; 5 INFN, Laboratori Nazionali del Sud, Via S. Sofia 62, 95123 Catania, Italy

**Keywords:** Natural hazards, Tectonics, Volcanology, Applied physics, Particle astrophysics

## Abstract

At Mount Etna volcano, the focus point of persistent tectonic extension is represented by the Summit Craters. A muographic telescope has been installed at the base of the North-East Crater from August 2017 to October 2019, with the specific aim to find time related variations in the density of volcanic edifice. The results are significant, since the elaborated images show the opening and evolution of different tectonic elements; in 2017, a cavity was detected months before the collapse of the crater floor and in 2018 a set of underground fractures was identified, at the tip of which, in June 2019, a new eruptive vent started its explosive activity, still going on (February, 2020). Although this is the pilot experiment of the project, the results confirm that muography could be a turning point in the comprehension of the plumbing system of the volcano and a fundamental step forward to do mid-term (weeks/months) predictions of eruptions. We are confident that an increment in the number of telescopes could lead to the realization of a monitoring system, which would keep under control the evolution of the internal dynamic of the uppermost section of the feeding system of an active volcano such as Mount Etna.

## Introduction

Mount Etna is a permanently active volcano that looms above the eastern coast of Sicily and lies upon a continental pedestal. The composition of its about 370 km^3^ of lava and pyroclastics, accumulated in the last 500 ka, is prevalently Na-rich hawaiitic and trachybasaltic^1^. The fault system through which the magma feeds the many eruptions of Mount Etna is characterized by extensional structures that are prevalently oriented NE–SW and NNW–SSE^2^. The Central Crater (CC) is the nodal point of such extensional tectonics, being placed at the intersection of these two fault systems, where the total extension is almost doubled. In the last hundred years the dramatic increase of the extension rate has produced, at the base of the CC, the opening of two sub-terminal sets of fractures, whose frequent eruptions have built up the North-East and South-East cones^3,4^ (respectively NEC and SEC). This assembly of three cones and a variable number of vents and fumaroles is known as the “Summit Craters” and it is the site of three types of volcanic activity: (a) emission of a persistent gas plume, even during non-eruptive periods, through fumaroles spotting the inner walls of the craters and sometimes through the free surface of the lava; (b) effusive eruptions with low emission rate (typically < 4 m^3^/s) and long duration (months/years), often occurring from short fractures at the base of SEC and NEC; (c) the third type of activity is represented by the paroxysms, short-lasting (hours) violent explosive eruptions occurring on the main vents of all craters, with tall lava fountains of up to 1 km high, fast lava flows and high emission rate (≈ 100 m^3^/s)^5^. In the last decades the summit paroxysms have become quite common, representing a notable volcanic hazard.

**Figure 1:**
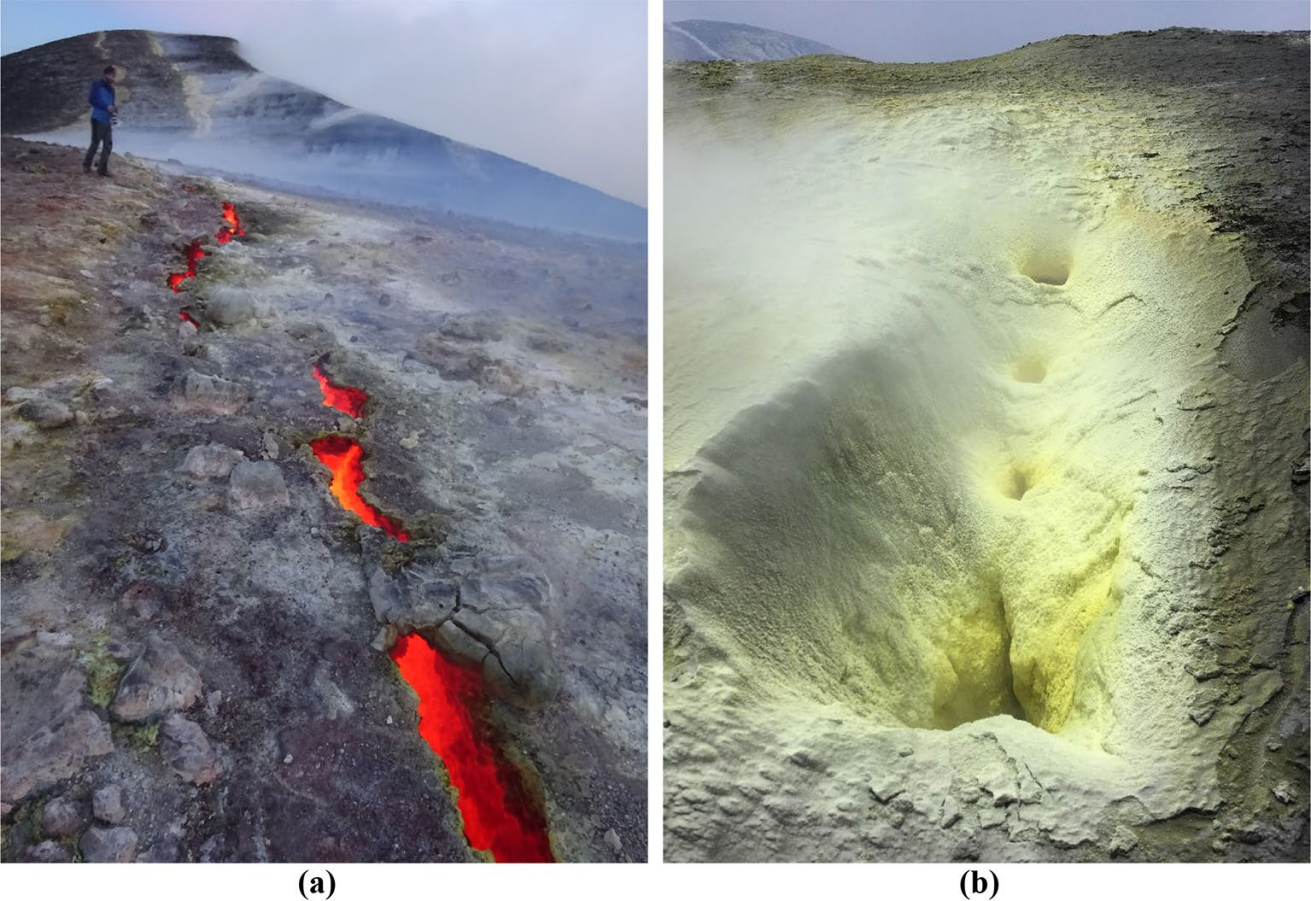
The surface expression of the fumarolic system (FS) on the Etnean summit craters. (**a**) Incandescent fracture on the top of the SEC; (**b**) sulfuric sublimates incrustating a degassing fracture at the northern flank of the NEC.

Permanently active volcanoes around the world such as Mount Etna or Stromboli are often defined as “open-conduit volcanoes”. However, though widely used, the meaning of this expression is not at all straight. In fact, in none of these volcanoes the conduit is really “open”. Direct observations indicate that during the eruption the conduit is dynamically occupied with the magma that is rising up; after the eruption the conduit is filled with a plug of cooled and solidified lava. The general picture we will use to describe the Etnean conduit or plumbing system has been recently drafted in^6^, where the last 2–3 km beneath the summit are described as vertically parted into three segments. The lowermost segment, that can be considered as the top of the deep plumbing system, is made of a pressurized, low density (1.14 t/m^3^), gas dominated magma; the intermediate segment, that is the shallow plumbing system, is constituted by a high density (2.8 t/m^3^), basaltic melt, through which the gas bubbles migrate to the surface; finally the uppermost segment, that we can define here as the “fumarolic system” (FS) is made by solidified, rigid lava, broken by multiple fractures, through which the permanent degassing occurs, as shown in Fig. 1.

The vertical extension and the volume of each of these three segments is not stationary but, on the contrary, is extremely erratic, depending on the fluid flux regime (that might evolve up to produce eruptive events), by the regional tectonics and by the evolution of the volcanic edifice itself. The comprehension of the plumbing system is a key element in the volcano dynamics and fundamental to do mid-term (weeks/months) predictions of volcanic eruptions.

Up to date, the study of the plumbing system is achieved through geophysical data: deep-seated earthquakes can provide information on the movements of main fault systems; ground deformations are used to detect the inflations or deflations of the volcanic edifice as a whole or in specific areas such as the rift zones; the geochemical monitoring provides information on the increments or decrements of the gas flux^7^. However, there are no monitoring devices right above the Summit Craters and therefore, the evolution of the FS is still unclear. If the plumbing system can be considered as a thermodynamic device, constituted by high temperature gas and molten basalt, whose function is convecting fluids upwards and releasing telluric heat into the atmosphere, it can be easily understood how this last, more rigid segment of the feeding system, would represent the “lid”, yet the weak link, of the entire structure. In other words, it could be of paramount importance to detect in due time the opening of new fractures or the enlargement of the existing ones to form bell-shaped cavities. The fracture opening, besides making the escape ways for fluids (primarily gas and subordinate molten basalt), could trigger, due to depressurization, the uprise of the gas rich portion of the magma, thus promoting violent eruptive paroxysms.

The FS represents a “blind spot”, a vulnerability of volcano monitoring systems exclusively based on traditional techniques. The muon radiography technique is very suitable to the investigation of the shallow level of the volcanic edifice, i.e. the portions of the structure which high energy cosmic muons can cross, which is, at the same time, the more difficult region to inspect by means of traditional or novel geophysical techniques.

Atmospheric muons are copiously produced by the interaction of highly energetic primary particles (mostly protons) with the atmosphere of our planet. Their flux, energy spectrum, angular distribution and interaction with matter (energy loss, multiple scattering, etc.), are reasonably well known^8^, allowing the use of secondary muons as a probe to investigate many aspects of the world around us^9^, from the search of hidden chambers in the Egyptian pyramids^10,11^ to imaging the content of cargo containers^12–16^, or even the monitoring of large civil structures^17,18^. X-rays or neutrons are used for medium size objects, but for larger objects the attenuation of such probes would be too high. On the contrary, cosmic muons can cross a significant thickness (hundred meters or even km) of solid material, such as mountains or volcanoes. The first proposal of studying the inner structure of a volcano using muons as a probe belongs to Nagamine et al. in 1995^19^. In 2003, Tanaka and collaborators produced the first muon radiography of a volcano in which the structure of the central crater was recognized^20^. This experiment was the proof that muography can obtain results to complement measurements by micro-gravimetry and resistivity. Since that moment, many successful applications of muon imaging followed^21–27^.

Muon radiography requires a particle detection apparatus, usually called “muon-telescope”, which reconstruct muon trajectories inside its field of view in order to infer their path through the studied object. Such a detector allows to measure the flux of muons traversing a mountain and the comparison with the corresponding flux measured from the open sky, i.e. without attenuation, will provide quantitative information on the absorption properties of the structure being probed. Results may be interpreted in terms of the opacity, evaluated as the density integrated along a path length, ${\text{O}} = \smallint \rho ({\text{x}}){\text{dx}}$, according to the well known concept of “radiography”, which is widely known for the X-ray images of the human body. This technique is usually referred to as transmission muography, because the image appears while crossing muons add-up. Since the angular distribution of atmospheric muons roughly follows a cos^n^ θ dependence on the zenithal angle θ, where n ≈ 2 (slightly dependent on the muon energy and geographical location), the flux of nearly horizontal muons is much reduced with respect to the vertical ones, thus requiring a long time exposure to get statistically significant results. The muon flux that crosses a volcano is affected by the variation of density encountered along their path. Once fixed the traversed thickness, the decrease of the muon flux, compared to the flux measured from the open sky, can directly be related to the average density along the chosen path. The resulting muography is therefore comparable to a 2D image of the rock density, where the colour scale is proportional to the average density.

The Muography of Etna Volcano (MEV) project started in 2016 and the collaboration involves physicists, engineers and volcanologists, which ensure a multi-disciplinary approach to the problem. Here, the results of the first two measurement campaigns at Mount Etna are presented.

## Results

### Site and measurements

A muon telescope, as employed in the present investigation, is aimed at detecting time related variations of the average density. In fact, within the FS (200–400 m from the crater edge) the variation of density of the volcanic rock is produced by the internal extensional dynamics, that is the time evolution of opening or closing during time of fractures that have usually meter size. Once a fracture is open, it is filled with high temperature volcanic gas, which has a very low density compared to surrounding basaltic rock. This will produce an increase of the muon flux detected by the telescope. These structural variations are not visible at the surface and to follow their evolution is very important.

**Figure 2:**
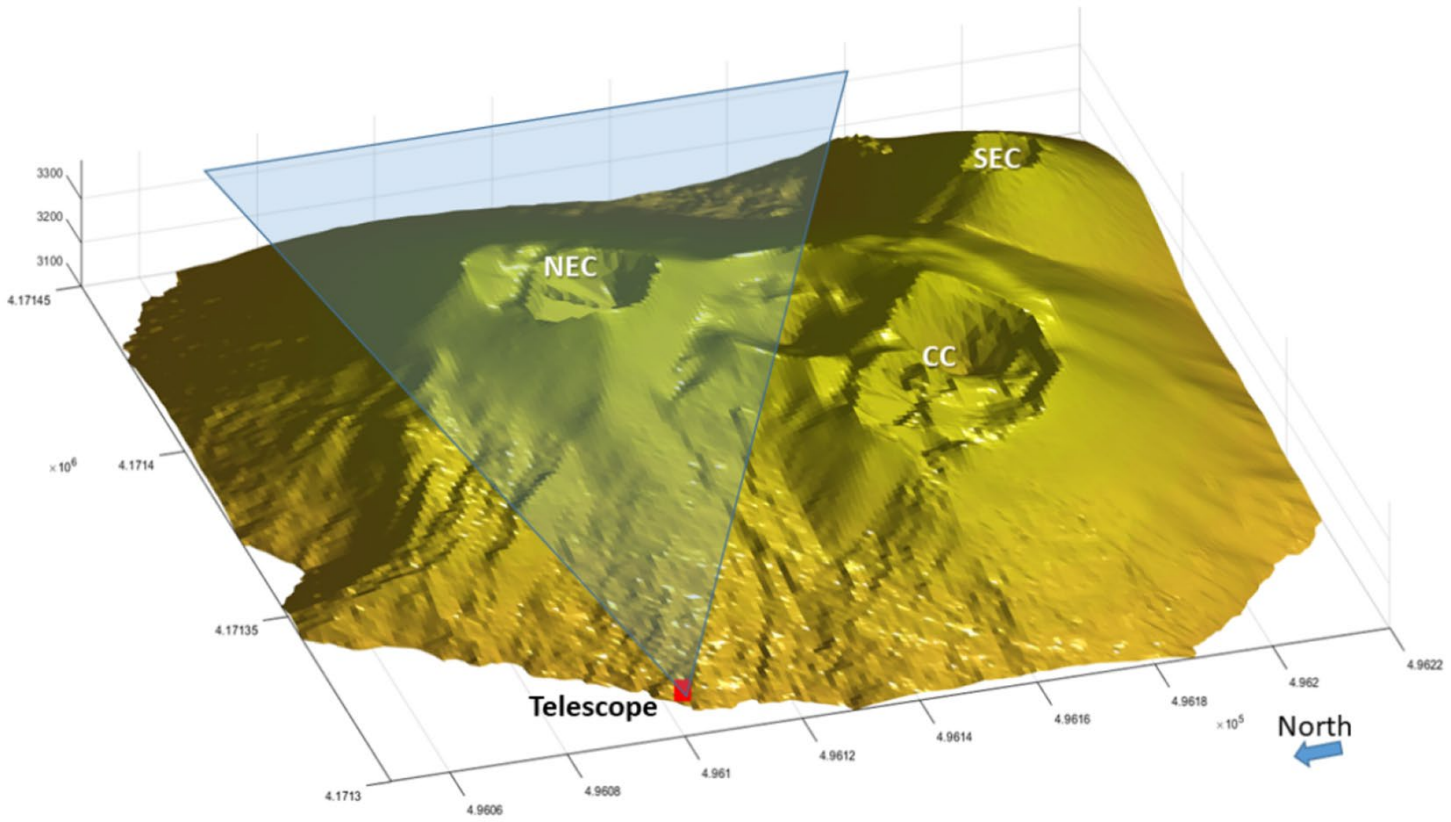
Position and field of view of the telescope, as installed at 3,100 m a.s.l. at about 700 m from the Etna NEC axis. This 3D view is obtained from a Digital Elevation Model (DEM), i.e. a X–Y–Z profile of the mountain site with a resolution of 10–20 m, acquired in 2014. Courtesy of National Institute of Geophysics and Volcanology (INGV), Etnean Observatory.

**Figure 3:**
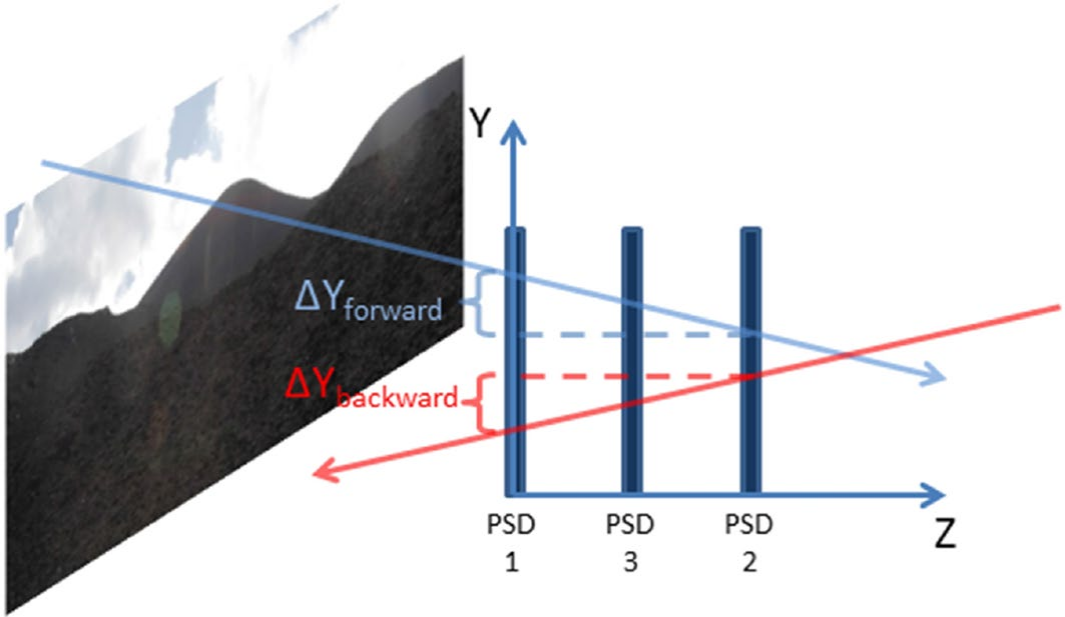
A sketch of the MEV telescope, made by three position sensitive detectors (PSD), segmented into X and Y oriented scintillating strips, such as to reconstruct the impact point of the muon track on the detection planes and the particle trajectory. In the left part (not to scale), a view of Mount Etna NEC in front of the PSD1 (T1). In the sketch is clearly described as the difference $\Delta y$ between the entrance and exit coordinate in the external planes allows to distinguish tracks coming from the front (target side) or from the back (open-sky side) of the detector.

For the purpose of this work and due to the availability of a single telescope (a particle tracker built within the MEV collaboration^28^), the extension of the summit craters (> 5 km^2^) has imposed some logistic choices. We have chosen the NEC whose northern base at 3,100 m a.s.l. was easily reachable with a truck. In order that the telescope works properly in transmission mode, it is necessary that no other macroscopic object lies behind the target. Figure 2 shows how the telescope was oriented to detect the muon flux passing through the NEC conduit only. At the same time, in front of the opposite side of the detector, there was no obstacle to attenuate the secondary-cosmic muon flux and the un-attenuated, or “open-sky” flux, can be measured. The telescope collected data from August 1st, 2017 until October 7th, 2017, when the snow covered the solar panels. In July 2018 it was possible to start a new acquisition which lasted until November 21st, 2018. In order to better understand the results shown in the following, it is necessary to disclose some details about the telescope. The telescope can be thought as an imaginary parallelepiped where two X–Y position sensitive (PS) planes are placed on two opposite faces and the third detection matrix lies parallel in the middle. The outer planes are segmented into $N \times N$ pixels; the combination of all possible pixel pairs of outer matrices defines a set of (2*N* − 1)^2^ discrete directions of sight *r*_*m,n*_, with $m = i - k$, $n = j - l$, where $(i,\;j)$ and $(k,\;l)$ represent the $(x,\;y)$ pixel indices on each matrix. The direction r_0,0_ is normal to the PS planes and is parallel to the telescope axis, oriented from back to front and passing through its center. Our telescope is designed to work horizontally placed, so *r*_*0,0*_ corresponds to the horizontal direction headed towards the target. In reporting the following results, we follow the common practice to display muography data as a function of X and Y displacements, $\Delta x$ and $\Delta y$, between the entrance and exit coordinates of muon tracks in the telescope external planes. Assigning the label *T*_1_ to the tracking module closest to the target (front-side) and *T*_2_ to the opposite outer plane (back-side), the convention $\Delta x[y] = x_{1} [y_{1} ] - x_{2} [y_{2} ]$ is adopted. This means that $\Delta y > 0$ identifies particles coming from the detector front and, on the contrary, $\Delta y < 0$ refers to particles entering the detector from the back side. Figure 3 shows a sketch of the setup with the three logical X–Y detection planes *T*_1_, *T*_2_ and *T*_3_ labeled as PSD1, PSD2 and PSD3, respectively.

**Figure 4:**
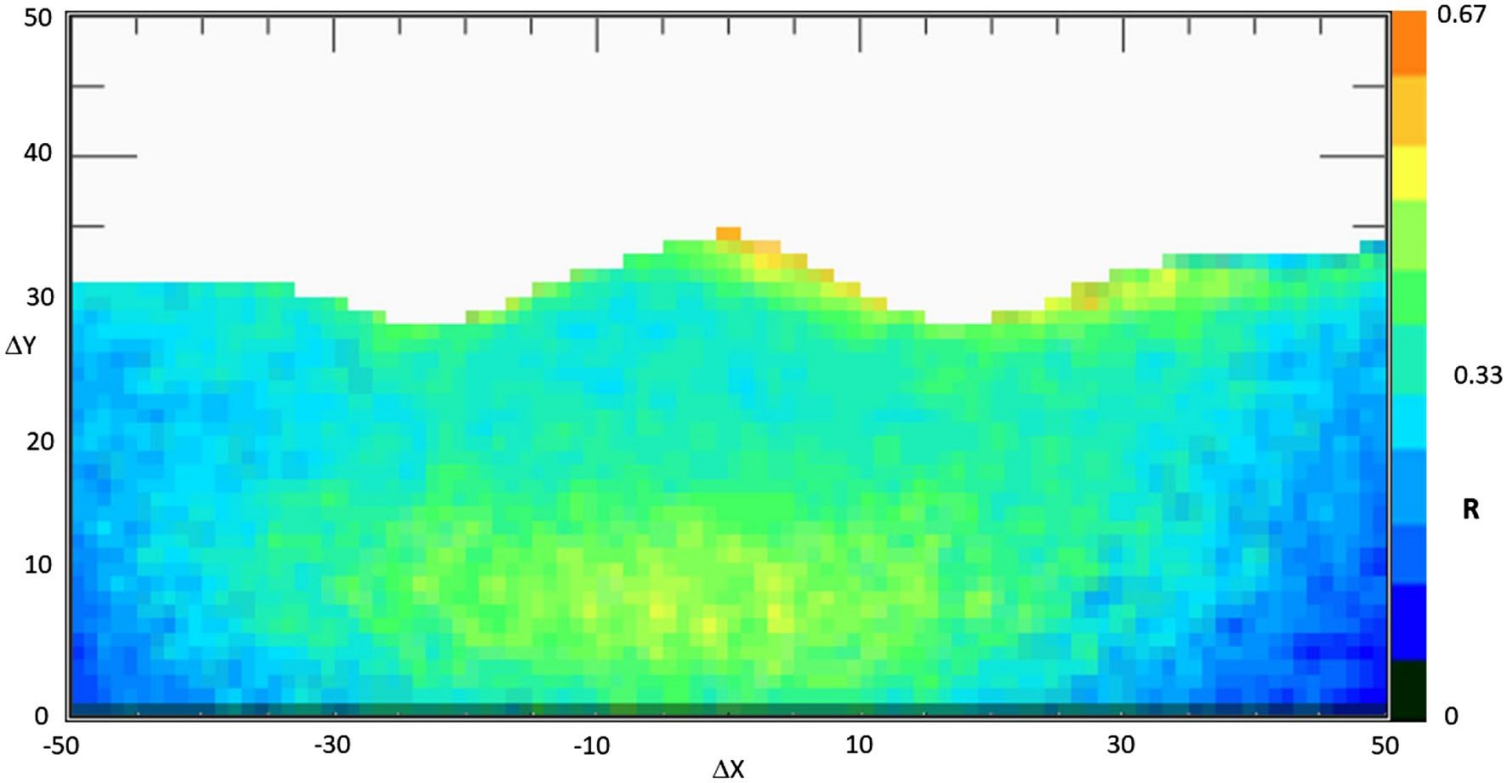
2017 muography of Etna volcano North-East crater. The image shows a map of the ratio *R* of muon flux through the mountain to that coming from the open-sky, i.e. without attenuation. *R* values are displayed as a function of X and Y displacements, *Δx* and *Δy*, between the entrance and exit coordinates of muon tracks in the telescope external planes.

**Figure 5:**
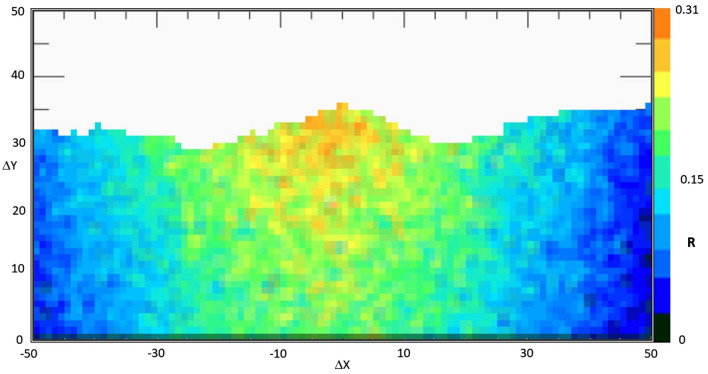
2018 muography of Etna volcano North-East crater. The image shows a map of the ratio *R* of muon flux through the mountain to that coming from the open-sky, i.e. without attenuation. *R* values are displayed as a function of X and Y displacements, *Δx* and *Δy*, between the entrance and exit coordinates of muon tracks in the telescope external planes.

Figures 4 and 5, corresponding to the two measurement periods, show the muographic results, represented as plots of the ratio *R* between the muon flux from the mountain side and the corresponding flux from the open sky, as a function of the horizontal and vertical displacements $\Delta x$ and $\Delta y$. This corresponds to assuming the detector as a point-size object with respect to the target. If the images are projected on a vertical plane, parallel to telescope position sensitive planes and passing through NEC axis, with this telescope geometry and its distance from the target, each pixel corresponds to a square of about 7 m side.

The colour scale in the muographies could be easily interpreted as follows: since the measured muon flux entering the telescope from the front side is affected by the attenuation due to the target object, we expect that the values of *R* range from 0 to 1, which correspond to a complete absorption and a complete transmission of the particles through the target, respectively. Once fixed the density, which can be assumed as the average value of standard volcanic rock (≈ 2.6 g/cm^3^), as the traversed thickness of rock increases, *R* tends to zero, while as the average density along the path diminishes *R* tends to unity. To focus on the high-density region (in the sky region the ratio of the fluxes is about 1), the sky over the profile of the mountain in the muography was excluded. For each pixel the combined effect of changes in thickness and average density modulates the colour in the linear colour scale.

## Discussion

In the central lower portion of Fig. 3 (− 15 < *Δx* < 15, 5 < *Δy* < 15, *R* nearly equal to 0.45–0.5), the ratio *R* is high with respect to the regions around, and being the thickness almost constant in this region we can conclude that this higher muon flux is due to a decreased cumulative density along the traversed path. Note that this value is similar to what is recorded in the top-most region of NEC, where the traversed thickness is lower, and the cumulative density is equal to that of standard rock. This can be interpreted as if, 200 m below the edge of the NEC, there was during the fall 2017 a set of large fractures or a bell-shaped cavity.

**Figure 6:**
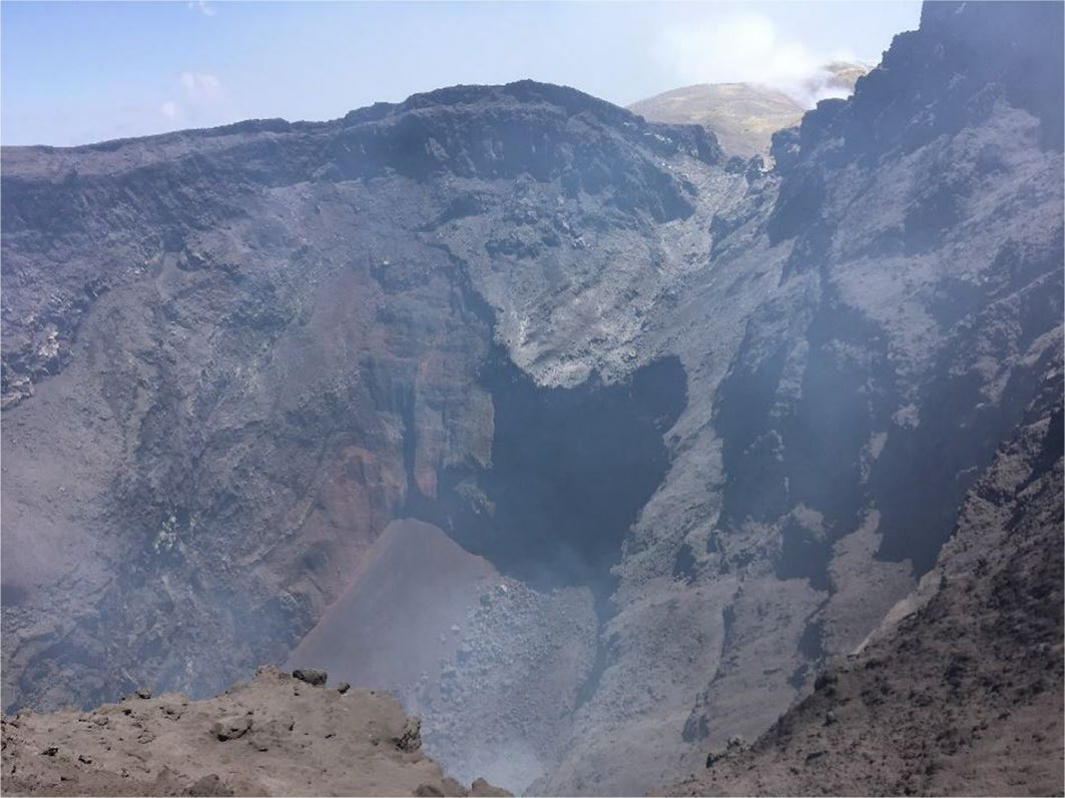
Picture of the inner flanks of the NEC, taken in December 2017. In the centre of the picture the remnants of the crater floor before the collapse are visible.

As a matter of fact, in December 2017, a collapse of the NEC floor occurred (Fig. 6). In our interpretation, the collapse was due to high temperature gases which rose from the inner part of the volcano and corroded slowly the upper structure, until it collapsed.

**Figure 7:**
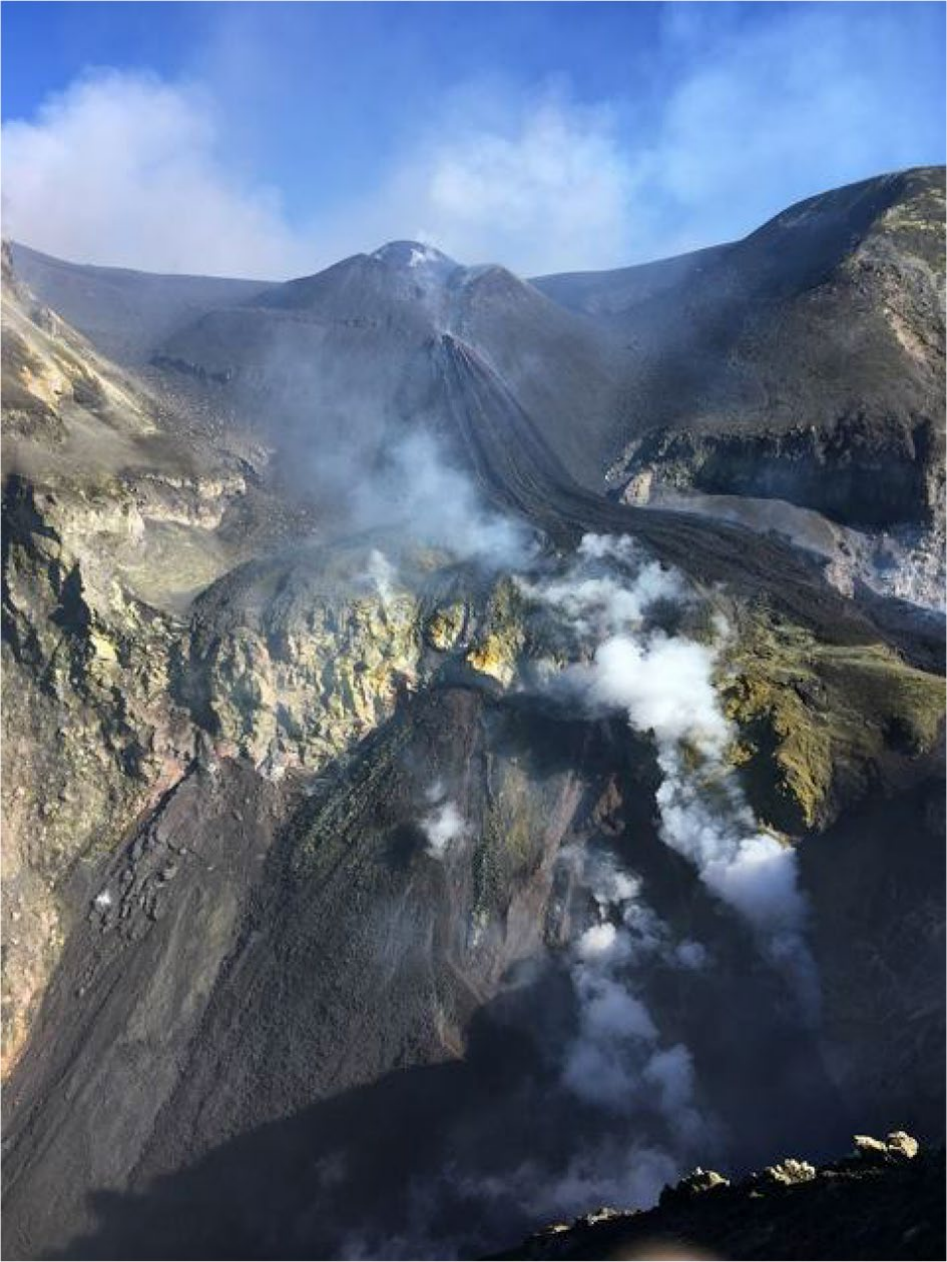
Picture of the CC in which is visible the pyroclastic cone and the lava flow, still in evolution (February 2020).

Moreover, Fig. 4 showed that about 50 m below the edge of the NEC the muon flux through the upper region of the NEC was high. In this case, the interpretation may be given in terms of the fractures that migrated from the NEC towards the CC. In fact, at the tip of this set of fractures, on the inner flank of the CC, see Fig. 7, the opening of a pit crater occurred, which began to erupt in June 2019 and is still erupting (February 2020).

After this successful interpretation, the second muography, Fig. 5, showed noticeable variations. In particular, it is evident a large difference in the ratio *R* (with values close to the maximum of the colour scale) in the sommital region, due to the combination of the floor collapse and fractures opening.

In conclusion, we can assert that the study of cosmic rays presents an intriguing outcome concerning the possibility to investigate the inner conduit of an active volcano such as Mount Etna. The feeding system of a volcano is controlled by the tectonics that is dynamically changing through time. In order to monitor such changes, we have built and installed a muographic telescope at the base of the North-East Crater (NEC) from August 2017 to October 2019. This was a pioneer experiment and was carried on with limited resources and only one telescope. We retain that the acquisition of muography data from at least two perspectives and their combination with data from other geophysical techniques are mandatory in order to perform a density estimation of the volcano internal structure. Even with these limitations, the results obtained are extremely good: we have observed in advance the opening of fractures that led to the collapse of the floor inside the NEC; we observed the fracture migration from the NEC towards the CC, which preceded the opening of an eruptive vent still going on (February 2020). In other words, the hypotheses we made about the volcanologic and tectonic interpretation of muography results have been confirmed by subsequent visual evidence. These results represent a solid basis in our project which foresees the realization of a permanent monitoring system of Mount Etna plumbing system by creating a network of muon telescopes for each Summit Crater, to be integrated with the existing infrastructures of Etnean Observatory by Italian National Institute of Geophysics (INGV). This might be of paramount importance to predict volcanic eruptions and to provide information to the civil protection authorities.

## Data and methods

### MEV muon-tracking detector

**Figure 8:**
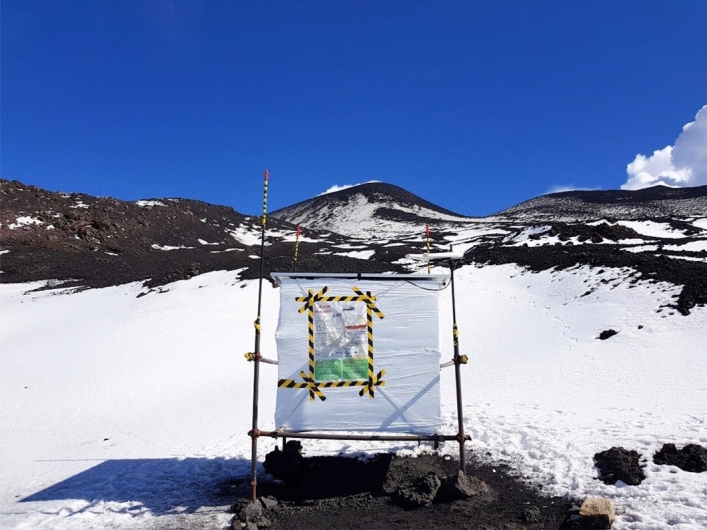
The MEV telescope placed on the slope of Etna NE crater in October 2018 during the second data taking campaign.

The first telescope developed for the MEV project was built at the Department of Physics and Astronomy of the University of Catania. The detector was designed to meet all the requirements of a long-term measurement campaign at the summit zone of the Etna Volcano. It is based on three X–Y position-sensitive planes, with a sensitive area of 1 m^2^. The tracking modules are enclosed in a cubic box with an external side of about 1.5 m, made with panels constituted by a double metallic cover and an inner isolating filling of polyurethane. The external box has been tested to be watertight and working as a dark box. The power supply of the detector is provided by two solar panels, mounted on the upper side of the external box, which charge a battery pack housed inside. The box is mounted on a modular frame in order to facilitate the transportation of the whole detector (weight about 300 kg) by means of a truck with a mechanical arm and the frame lies on adjustable legs to cope with uneven terrain. However, the inner structure of the telescope is fully modular and can be assembled on field, even without the external box for a short data taking campaign during summer. Figure 8 shows the telescope installed at the measurement site at about 3,100 m a. s. l. at the Etna NE crater.

Each sensitive module consists of two layers of *N* = 99 extruded plastic scintillator bars made by Fermilab^29^ (nominally 1 × 1 × 100 cm^3^) with a central 2.5 mm hole inside through which two 1 mm Wavelength Shifting (WLS) fibres (Saint-Gobain BCF-92)^30^ are embedded to transport the photons to a multi-anode photomultiplier (MAPMT). The plastic scintillator is coated with a white reflector on each external side. More details about muon tracks reconstruction procedure and related flux measurement with the MEV telescope are discussed in^31^. Each PS plane has 2 × *N* intersecting strips, and, therefore, 4 × *N* optical channels, one for each WLS. The overall number of read-out channels is 3 × (4 × *N*) = 1,188, but this number has been minimized by a factor $1/\sqrt N$ by means of a channel reduction system already employed in other tracking detectors^16,32^. The two WLS in each strip, are routed separately in a *Group*-set and in a *Strip*-set. A *Group*-set brings together the WLS of *n* adjacent strips, while in a *Strip*-set the *i*-th strip of each *Group* is routed. In this way, the strip hit by a particle can be unambiguously reconstructed by the equation *Strip*_*hit*_ = (*i* − 1) × *n* + *j,* where *i* corresponds to the *i*-th *Group*-set and *j* stands for the *j*-th *Strip*-set.

**Figure 9:**
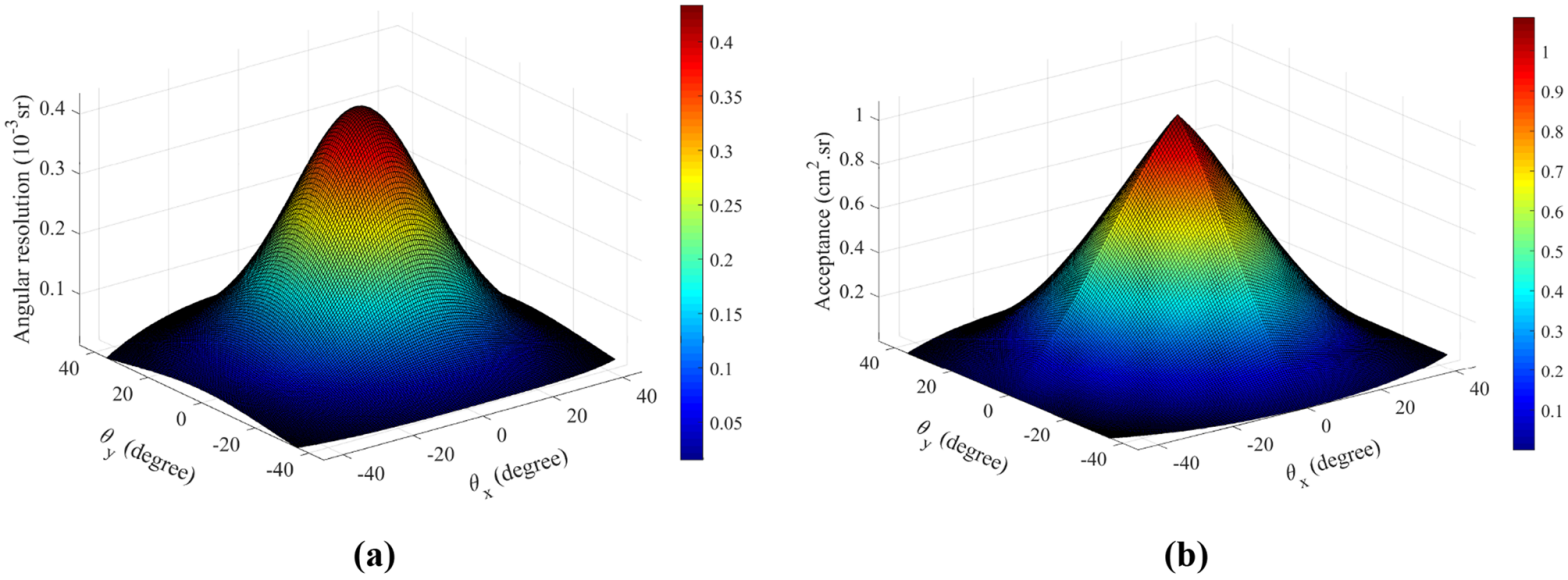
Distribution of the angular resolution $\delta \Omega$ (**a**) and of the Acceptance ${\mathcal{T}}$ (**b**) for each discrete direction of site of the telescope, displayed as a function of azimuth $\theta_{x}$ and zenith $\theta_{y}$ angles corresponding to each *r*_*m*,*n*_.

The number *N* of pixels in outer PS planes, their size *p* and the distance *D* between front and back matrices establish the total solid angle covered by the telescope and its angular resolution. Figure 9a shows the angular resolution $\delta \Omega (r_{m,n} )$ of the telescope employed for the measurement discussed in this work: with *p* = 100/*N* cm and *D* = 97 cm, the angular aperture is about ± 45° and the angular resolution has a maximum nearly equal to $\delta \Omega \;(r_{0,0} ) \simeq 4.3 \times 10^{ - 4}$ sr. By grouping together all the pixel couples, one for each external matrix, which share the same direction *r*_*m,n*_—or, equivalently, the same $(\Delta x,\;\Delta y)$—the detection area associated with a direction of sight depends on the number of pixel pairs. The full set of geometric characteristics of the telescope are summarized by the acceptance function ${\mathcal{T}}(r_{m,n} )$, given in cm^2^ sr as defined in^22^, and shown in Fig. 9b.

The electronic chain is fully custom designed for the purpose of the MEV project in order to have low power consumption. It can be divided into two main parts, the Front-End (FE) and the Read-Out (RO) electronics. The FE consists of three boards, one for each PS module. Each FE board houses a Hamamatsu H8500 MAPMT with 64 channels^33^ and a MAROC3 chip^34^. In the pre-processing phase, the chip pre-amplifies and shapes the analog signal from the MAPMT with a peak time of about 20 ns and compares all the signals to a common threshold, giving a digital time-over threshold signal for each channel.

The output from each FE board is then acquired, filtered and processed by a National Instrument SOM (System-on-Module)^35^ mounted on a single RO board, which also houses the sensing components for temperature and movement (accelerometer). The SOM allows a graphical approach for programming its FPGA with LabVIEW. A dedicated User Interface (UI) has been developed to manage and control all the parameters of the system.

**Figure 10:**
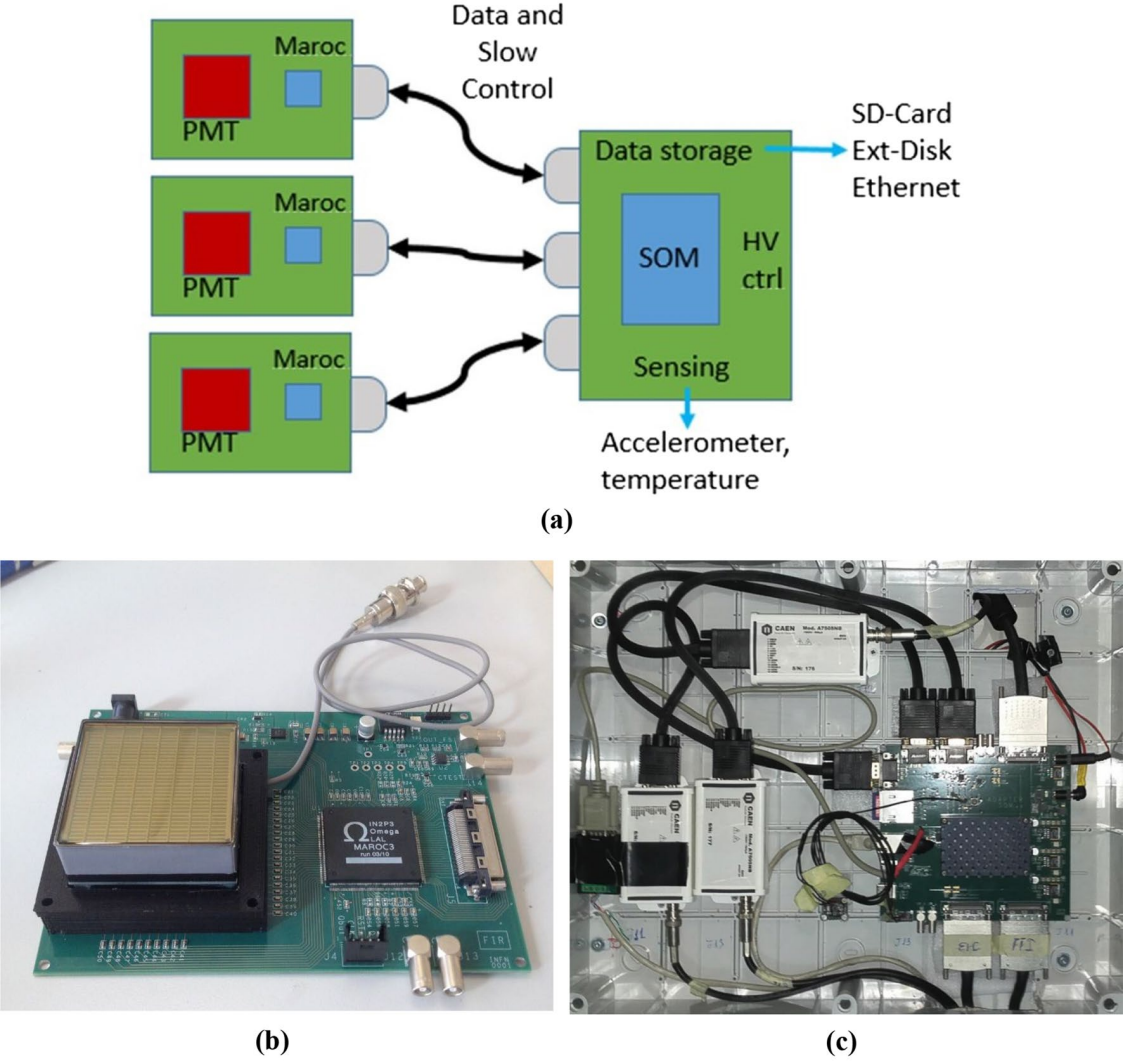
(**a**) Block diagram of the electronic chain of the telescope. (**b**) A front-end board equipped with a Hamamatsu H8500 64 channels MAPMT. (**c**) A photo of the read-out board placed inside a plastic box with the modules for MAPMTs power supply.

A scheme of the complete electronic chain is sketched in Fig. 10, together with pictures of FE and RO boards.

Data is stored in a local SD card memory. The acquisition chain is set to write a bunch of data in a separate file every 5 min (ten during the 2017 campaign). The system is equipped with a 4G LTE wireless router. When the network signal is available, the data are sent to a cloud storage at the Department of Physics at a rate depending on signal strength.

### Data analysis

The quantity *R* shown in “Results” section, Figs. 4 and 5, can be formally defined as:

$$R = \frac{1}{t}\mathop \sum \limits_{i = 1}^{n} r_{i}$$

where $r_{i}$ is the ratio between the integral muon fluxes from the target-faced side of the telescope and that from the open-sky side, measured within a day, and *t* is the effective measurement time, expressed as a fraction of nominal days of acquisition. Usually, the duty cycle is 100%. We occasionally performed some calibration procedures so this is taken into account. In calculating the daily ratio *r*_*i*_, the pixels in which no counts from the open-sky side were registered, are excluded. Daily flux of muons coming from the front of the detector, which is in part reduced by the mountain, and that coming from the opposite side, without any attenuating agent in front, are measured at the same time and compared in calculating the ratio above defined. The muon azimuth angular distribution measured with the MEV telescope has been investigated^36^ and a correction for compensating the East–West effect near the top of Mount Etna is included in the calculation, even if it could be neglected in first approximation and flux could be considered isotropic with respect to the azimuth (horizontal angle).

Methods for data analysis and particle tracks reconstruction are now completely refined, so that for the future it will be possible to include them in a system monitor to display muographic images day by day. This will be helpful in order to provide an alert system for noticeable opacity variations which could trigger a volcanic eruption in a short-mid period.

## Data Availability

The datasets generated during and/or analysed during the current study are available from the corresponding author on reasonable request.
